# Phenolic Composition, Antioxidant Properties, and Inhibition toward Digestive Enzymes with Molecular Docking Analysis of Different Fractions from *Prinsepia utilis* Royle Fruits

**DOI:** 10.3390/molecules23123373

**Published:** 2018-12-19

**Authors:** Xuan Zhang, Yijia Jia, Yanli Ma, Guiguang Cheng, Shengbao Cai

**Affiliations:** 1 Yunnan Institute of Food Safety, Kunming University of Science and Technology, Kunming 650500, China; twinklezx65@163.com (X.Z.); yijia529@163.com (Y.J.); chengguiguang@163.com (G.C.); 2 College of Food Science and Technology, Hebei Agricultural University, Baoding 071001, China; xuexi_myl@sina.com

**Keywords:** hydrogen bond, oxidative stress, phenolic composition, pancreatic lipase inhibition, α-glucosidase inhibitor, UHPLC-ESI-HRMS/MS

## Abstract

The present study investigated the phenolic profiles and antioxidant properties of different fractions from *Prinsepia utilis* Royle fruits using molecular docking analysis to delineate their inhibition toward digestive enzymes. A total of 20 phenolics was identified and quantified. Rutin, quercetin-3-*O*-glucoside, and isorhamnetin-3-*O*-rutinoside were the major phenolic compounds in the total phenolic fraction and flavonoid-rich fraction. The anthocyanin-rich fraction mainly contained cyanidin-3-*O*-glucoside and cyanidin-3-*O*-rutinoside. All of the fractions exhibited strong radical scavenging activities and good inhibition on cellular reactive oxygen species (ROS) generation in H_2_O_2_-induced HepG2 cells, as evaluated by DPPH and 2,2′-azino-bis (3-ethylbenzothiazoline-6-sulphonic acid) (ABTS) assays. Moreover, the powerful inhibitory effects of those fractions against pancreatic lipase and α-glucosidase were observed. The major phenolic compounds that were found in the three fractions also showed good digestive enzyme inhibitory activities in a dose-dependent manner. Molecular docking analysis revealed the underlying inhibition mechanisms of those phenolic standards against digestive enzymes, and the theoretical analysis data were consistent with the experimental results.

## 1. Introduction

In recent years, incidences of diseases related to metabolic syndrome have risen rapidly around the world, such as obesity, cardiovascular disease, and diabetes [1]. A high-calorie diet and oxidative stress are considered to play important roles in the incidence and development of metabolic syndrome [2,3]. Although, to some extent, some chemical drugs have been exploited to alleviate or treat these diseases, the side effects of these drugs may cause profound threats to people’s health. Therefore, how to prevent or alleviate these diseases with few side effects has become a hot topic for scientists. A number of epidemiological studies have revealed that a diet rich in vegetables and fruits can effectively prevent or alleviate metabolic syndrome [4,5,6]. Food scientists and nutritionists have conducted many substantial studies in order to unveil the underlying reasons for the health benefits that vegetables and fruits exert. It is found that some phytochemicals in the plant-based foods may play important roles in the prevention and/or mitigation of metabolic syndrome-related diseases, such as phytosterols and phenolics [7,8].

Plant phenolics, a group of plant secondary metabolites, are characterized by the presence of an aromatic ring containing one or more hydroxyl substituents that are widely distributed in plants [6]. According to the structure, phenolics are divided into many subclasses, including phenolic acids, flavonoids, and anthocyanins, among others [9]. These metabolites not only play very important roles in plants to protect them against various physical and chemical damages, such as plant diseases, insect pests, and ultraviolet injury, they also have profound effects on the health of human beings. Epidemiological, clinical, and nutritional studies strongly support the evidence that plant phenolic compounds enhance human health by reducing risks of various diseases, including cancers, cardiovascular diseases, degenerative diseases, and metabolic disorders [7]. Previous studies have pointed out that the various physiological bioactivities of plant phenolics are partially related to their antioxidant properties [10]. Moreover, some literatures have also reported that phenolic-rich extracts from various plant materials exhibited good inhibitory effects on digestive enzymes, e.g., α-amylase, α-glucosidase, and pancreatic lipase [11,12,13,14]. Therefore, for health concerns, phenolic-rich vegetables and fruits are recommended for daily consumption by food scientists and nutritionists as natural inhibitors of digestive enzymes to reduce fat and glucose uptake.

*Prinsepia utilis* Royle, which belongs to the Rosaceae family, is a deciduous perennial shrub. This plant mainly grows in high-altitude areas (1000–2500 m) in northern India and southwest China in the Himalayan mountain district [15]. *P. utilis* is an economic tree in that it requires undemanding soil conditions and is commonly found on uncultivated land. In Chinese and Indian folk medicine, all parts of this plant, such as the roots, leaves, seeds, and fruits, have long been used to treat numerous diseases, such as rheumatism, skin diseases, and fractures [16,17]. Indigenous people have traditionally considered the seeds of this plant a food material and an essential component for oil extraction. Several phytochemical studies have been conducted to isolate and identify the natural products in *P. utilis*, showing that this plant contained hydroxynitrile glucosides, triterpenoids, and diterpene glucosides [16,17,18]. However, information on the phenolic profile and bioactivities of *P. utilis* fruits is extremely scarce, thus hindering the further development of the commercial utilization of *P. utilis*. Therefore, this study aims to comparatively investigate the phenolic compositions and antioxidant properties of different fractions from *P. utilis* fruits; meanwhile, their inhibitory effects against α-glucosidase and pancreatic lipase were also evaluated and analyzed by molecular docking assay. The present work may provide related data for further study and the utilization of *P. utilis* in functional food and nutraceutical industries.

## 2. Results and Discussion

### 2.1. Characterization and Quantification of Phenolic Compounds

Phenolic compounds, as a main kind of secondary metabolite in plants, consist of several subcategories, including phenolic acids, flavonoids, and anthocyanidins, among others [19]. These compounds not only play key roles in plants to protect them against biotic and abiotic hazards [20], they also have very important bioactivities for human beings to improve health conditions [21]. Dietary phenolic compounds mainly come from various fruits and vegetables, and they are often considered the primary bioactive substances in those fruits and vegetables [22]. 

**Figure 1:**
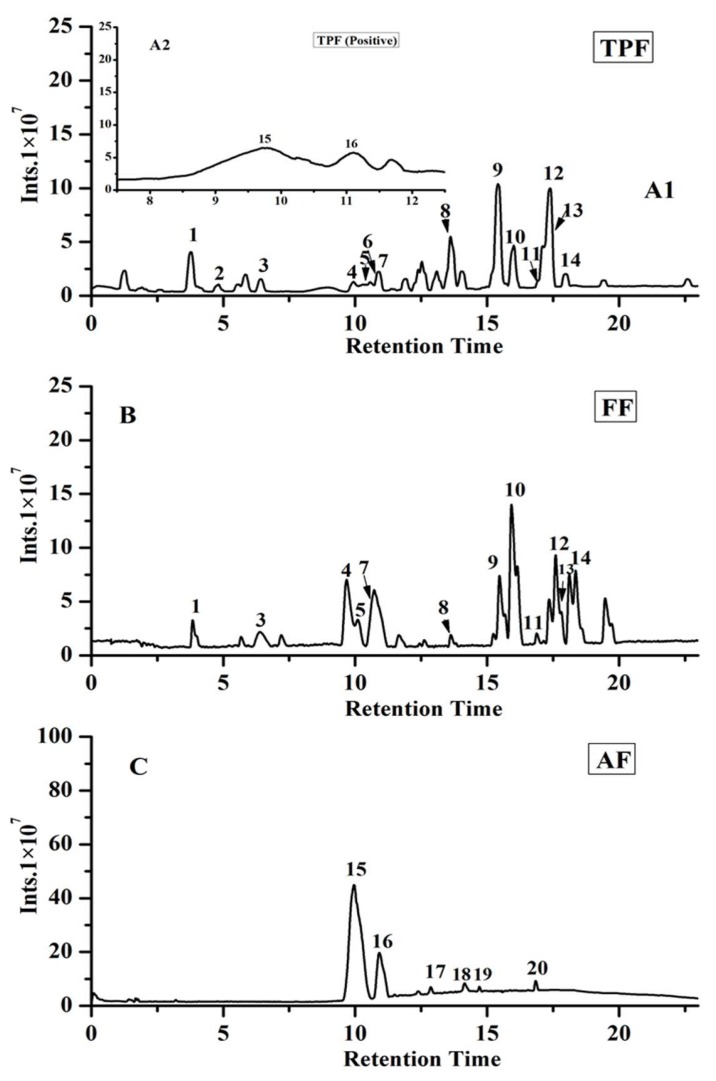
Negative (**A1**,**B**) and positive (**A2**,**C**) ion current chromatograms of different fractions from *P. utilis* fruits: total phenolic fraction (**A**), flavonoid-rich fraction (**B**), anthocyanin-rich fraction (**C**). The identification of peaks and their MS data are shown in Table 1.

In the present work, the phenolic compositions of total phenolic fraction (TPF), flavonoids fraction (FF), and anthocyanin fraction (AF) from *P. utilis* fruits were tentatively or positively identified by UHPLC-ESI-HRMS/MS in a negative mode (non-anthocyanin phenolics) or positive mode (anthocyanins). The ion current chromatograms are illustrated in Figure 1, and the related mass data, such as retention time (RT), *m/z*, error, molecular formula, and MS/MS ion fragments, are summarized in Table 1. In this work, the phenolic characterization was achieved by comparing their mass data with those of their authentic standards, or with the mass information reported in the literature or public mass database (namely, Massbank, http://www.massbank.jp/QuickSearch.html). As seen in Figure 1 and Table 1, a total of 20 compounds were detected and characterized, 19 of which were tentatively or positively identified as phenolic compounds, including two phenolic acids, 11 flavonoids and their derivatives, and six anthocyanins. For the TPF, compounds **9** and **12** (peaks **9** and **12**, respectively) were found to have the higher peak areas, suggesting that those two phenolics were the main compounds in the TPF extracts. Compound **9** ([M − H]^−^ *m/z* = 609.1476) was positively characterized as rutin by comparing it with the corresponding authentic standard. The characteristic ion fragment (*m/z* = 300.0282) of compound **9** resulted from the loss of rutinose moiety. Compound **12** ([M − H]^−^ *m/z* = 623.1636) was positively identified as isorhamnetin-3-*O*-rutinoside by a commercial phenolic standard. The loss of rutinose moiety of isorhamnetin-3-*O*-rutinoside produced a characteristic ion at *m/z* = 315.0519. However, compound **10** was detected with the highest peak area in FF, which was tentatively identified as quercetin-3-*O*-glucoside ([M − H]^−^ *m/z* = 463.0896), and the fragment ion (*m/z* = 300.0282) was produced from the loss of glucose moiety from compound **10**. Compound **15** ([M]^+^ *m/z* = 449.1074), which was positively characterized as cyanidin-3-*O*-glucoside, was the main phenolic compound detected in the AF: the loss of glucose moiety from compound **15** resulted in the production of a fragment ion with *m/z* = 287.0548. Most of the phenolic compounds that were detected in the present work, especially anthocyanins, have not been reported in this plant to date. However, protocatechuic acid, rutin, quercetin-3-*O*-glucoside, and isorhamnetin-3-*O*-rutinoside, all of which were detected with higher peak areas in the present research, were also reported by a previous work in the seeds of *P. utilis* [15].

The quantitative results of all of the phenolic compounds in those fractions are summarized in Table 2. In the current study, 10 of the identified phenolic compounds (**1**, **3**, **4**, **7**, **8**, **9**, **10**, **12**, **14**, and **15**), were quantified by the calibration curve of the corresponding authentic standard. The other nine detected phenols (**5**, **6**, **11**, **13**, **16**, **17**, **18**, **19**, and **20**) were quantified by the calibration curve of the standard shared a similar—aglycone. As seen in Table 2, the rutin and isorhamnetin-3-*O*-rutinoside were the predominant phenolic compounds in the TPF. However, quercetin-3-*O*-glucoside was found to have the highest content in the FF extract, which was isolated and purified from TPF. The rutin in FF decreased by half when compared with that in TPF, suggesting that the rutin in TPF extract may be lost or hydrolyzed during the subsequent isolation and purification. The contents of cyanidin-3-*O*-glucoside and cyanidin-3-*O*-rutinoside in AF (isolated and purified from TPF extract) were about 5.63 and 5.00 times higher, respectively, when compared to the corresponding anthocyanin in TPF. 

### 2.2. Antioxidant Capacity

Although the phenolic compounds possess many important bioactivities that have been reported by previous studies [27], antioxidant property is considered to be partly related to most of those bioactivities [28]. Therefore, antioxidant capacity in vitro was often used to evaluate the health benefit of a food material. Many different *in vitro* methods have been developed to measure the antioxidant capacity, such as chemical methods DPPH, 2,2′-azino-bis (3-ethylbenzothiazoline-6-sulphonic acid) (ABTS) and cellular antioxidant method (inhibition on intracellular reactive oxygen species, or ROS). In the current study, DPPH, ABTS, and intracellular ROS inhibition methods were applied to evaluate the antioxidant activity of the different fractions from *P. utilis* fruits. 

#### 2.2.1. DPPH Radical Scavenging Activity

**Figure 2:**
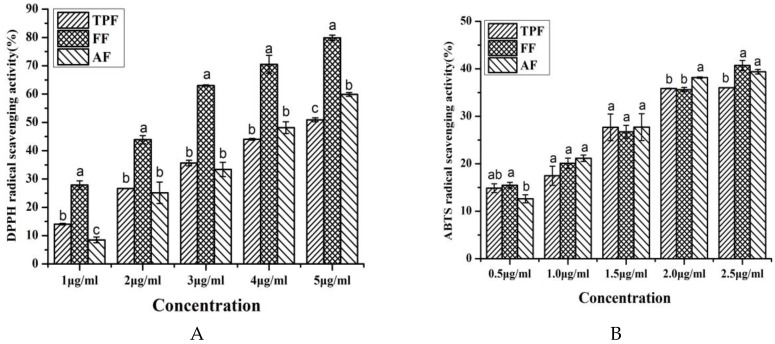
DPPH radical scavenging ratio (**A**) and 2,2′-azino-bis (3-ethylbenzothiazoline-6-sulphonic acid) (ABTS) radical scavenging ratio (**B**) of different fractions from *P. utilis* fruits. Values are showed as the mean ± SD (*n* = 3). Means with different letters at the same concentration are significantly different (*p* < 0.05).

The antioxidant activities of different fractions from *P. utilis* fruits evaluated by DPPH assay are presented in Figure 2A: All of the samples exhibited –DPPH radical scavenging activities in a concentration-dependent manner, especially the FF, which showed significantly stronger DPPH radical scavenging activity than the other two fractions at all of the tested concentrations, ranging from 1.0 μg/mL to 5.0 μg/mL (*p* < 0.05). The IC_50_ values of TPF, FF, and AF were 4.72 ± 0.31 μg/mL, 2.46 ± 0.11 μg/mL, and 4.20 ± 0.14 μg/mL, respectively. The IC_50_ value of Vc was 2.32 ± 0.11 μg/mL, which was similar to that of FF, but lower than those of TPF and AF. Among those three fractions, the FF exhibited the strongest DPPH radical scavenging activity (*p* < 0.05), which may be due to its highest phenolic content. A Pearson’s correlation analysis between the phenolic contents and DPPH radical scavenging activities proved that the phenolic contents of those three fractions in five different concentrations were closely related to their DPPH radical scavenging ratio (r = 0.976, *p* < 0.01), suggesting that the phenolic compounds contributed significantly to the DPPH radical scavenging capacity of different fractions from *P. utilis* fruits. The current result was consistent with many previous studies [12,15]. Huang et al. [15] reported that the phenolic compounds in *P. utilis* seeds were responsible for their DPPH radical scavenging activities, regardless of different pretreatments. Zhang et al. [13] also considered phenolics to be the main bioactive substances in *Rhus chinensis* Mill. fruits to scavenge DPPH radicals.

#### 2.2.2. ABTS Radical Scavenging Activity

The ABTS radical scavenging results of different fractions from *P. utilis* fruits are shown in Figure 2B. As seen in Figure 2B, all of the samples showed good and almost similar ABTS radical scavenging activities at the current concentrations in a concentration-dependent response. About 40% of the ABTS radicals were scavenged by TPF, FF, and AF just at 2.5 μg/mL, indicating that those fractions possessed ABTS radical scavenging activity. The IC_50_ values of TPF, FF, and AF were 3.45 ± 0.48 μg/mL, 3.2 ± 0.26 μg/mL, and 2.93 ± 0.35 μg/mL, respectively. Moreover, the IC_50_ value of Vc was 1.92 ± 0.21 μg/mL, which was lower than those of all three phenolic fractions in the present work. Phenolic compounds in *P. utilis* fruits may be also in charge of the ABTS scavenging capacities, which were confirmed by a high Pearson’s correlation coefficient between the phenolic contents and ABTS radical scavenging ratio (r = 0.889, *p* < 0.01). Some discrepancies between the IC_50_ values of DPPH and ABTS assays of the same fraction from *P. utilis* fruits were observed, which may be due to the different radical structure. Many previous studies also found a similar phenomenon when the DPPH results were compared with the ABTS findings [12,29].

In present work, rutin, isorhamnetin-3-*O*-rutinoside, and cyanidin-3-*O*-glucoside were identified as the major phenolic compounds in those fractions. A previous study reported that the rutin exerted good antioxidant activities, as evaluated by DPPH and ABTS radical scavenging assays with IC_50_ values of 29.63 μg/mL and 5.34 μg/mL, respectively [30]. Galati et al. [31] also observed that the juice of prickly pear (*Opuntia ficus indica* (L.) Mill.) fruits mainly containing isorhamnetin-3-*O*-rutinoside showed a good DPPH radical scavenging activity, with IC_50_ values of 6.75 μL, and the total phenolic compounds of this juice were 746 μg/mL. Sun et al. [32] also found that the anthocyanin extract from purple rice, which mainly had cyanidin-3-*O*-glucoside and other cyanidin derivatives, exhibited strong DPPH and ABTS radical scavenging activities, with IC_50_ values of 7.69 μg/mL and 7.11 μg/mL, respectively. The findings of those aforementioned studies further proved that the phenolic compounds in those fractions may contribute significantly to their antioxidant activities.

#### 2.2.3. Inhibition of Intracellular ROS in H_2_O_2_-Induced HepG2 Cells

**Figure 3:**
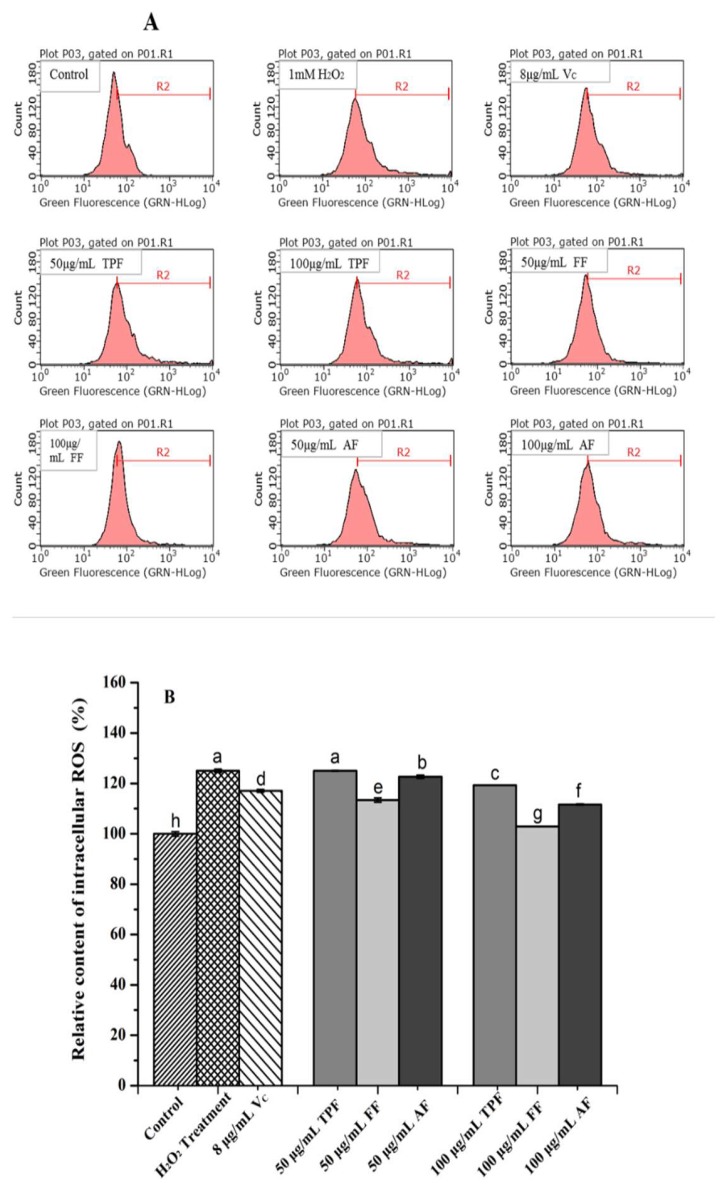
The results of cellular reactive oxygen species (ROS) inhibitory effects of the different fractions from *P. utilis* fruits in H_2_O_2_-induced HepG2 cells: Flow cytometry analysis (**A**) and the relative amount of ROS in different groups (**B**). Means (bar value) with different letters are significantly different (*p* < 0.05).

Normally, a certain amount of reactive oxygen species (ROS), as signal molecules, play important physiological roles in the body. However, the overproduction of ROS, which is often induced by various conditions (e.g., smoking, pollution, or other stress), may attack normal cells or biomacromolecules, resulting in tissue or organ damage, which is considered to be responsible for many disorders, such as age-related degenerative, cardiovascular, cancer, and other chronic diseases [33,34]. Therefore, the inhibitory effects of different phenolic fractions from *P. utilis* fruits on intracellular ROS generation in H_2_O_2_-induced HepG2 cells were evaluated, and the results are present in Figure 3: the intracellular ROS levels of HepG2 cells treated with H_2_O_2_ were significantly higher than those of the control group (*p* < 0.05), suggesting that the oxidative stress of HepG2 cells was successfully induced by one mM of H_2_O_2_. However, the ROS levels of HepG2 cells pretreated by 50.0 μg/mL and 100.0 μg/mL of different phenolic fractions dramatically decreased when compared to that of an H_2_O_2_ treatment group (*p* < 0.05), except for that of the 50.0 μg/mL of TPF pretreated group (*p* > 0.05). Among the three phenolic fractions, the FF exhibited the strongest inhibitory effect on intracellular ROS generation at each tested concentration, followed by AF. However, the TPF had the weakest ROS inhibitory activity (*p* < 0.05). More than 20% of intracellular ROS were inhibited in 100.0 μg/mL of the FF pretreated group when compared with that of the H_2_O_2_ pretreatment group. Moreover, a clear correlation between the phenolic contents of different fractions and their inhibitory activities was observed in the present work. In addition, 8.0 μg/mL of ascorbic acid (Vc) as a positive control also significantly inhibited intracellular ROS generation (*p* < 0.05).

Plant phenolic compounds have long been known for their outstanding antioxidant activity. Therefore, the intracellular ROS inhibitory effects of various phenolic compounds from different plant materials have been investigated extensively and deeply [35]. Zhuang et al. [35] reported that rambutan (*Nephelium lappaceum*) peel phenolics significantly inhibited the intracellular ROS generation of H_2_O_2_-induced HepG2 cells partly through upregulating SOD activity. Cilla et al. [36] also found that the bioaccessible, flavonoid-rich fractions of citrus fruit pulps dramatically decreased the intracellular ROS generation of Caco-2 cells induced by H_2_O_2_ [36]. Moreover, another previous study also pointed out that quercetin inhibited the intracellular ROS generation of H_2_O_2_-induced ARPE-19 cells via nuclear factor erythroid 2-like 2 pathway activation and endoplasmic reticulum stress inhibition [37]. Although the inhibitory effects of various plant materials on the intracellular ROS generation of H_2_O_2_-induced cells may be significantly different, all of the aforementioned studies pointed out that the phenolics contributed significantly to the ROS inhibitory activity, which was in agreement with the findings obtained in the present work. Moreover, previous study had found that polyphenols can up-regulate endogenous antioxidant enzymes or increase GSH content to protect cells against oxidative damage [38].

### 2.3. Pancreatic Lipase Inhibitory Activities of Phenolic Fractions from P. utilis Fruits and Authentic Standards

**Figure 4:**
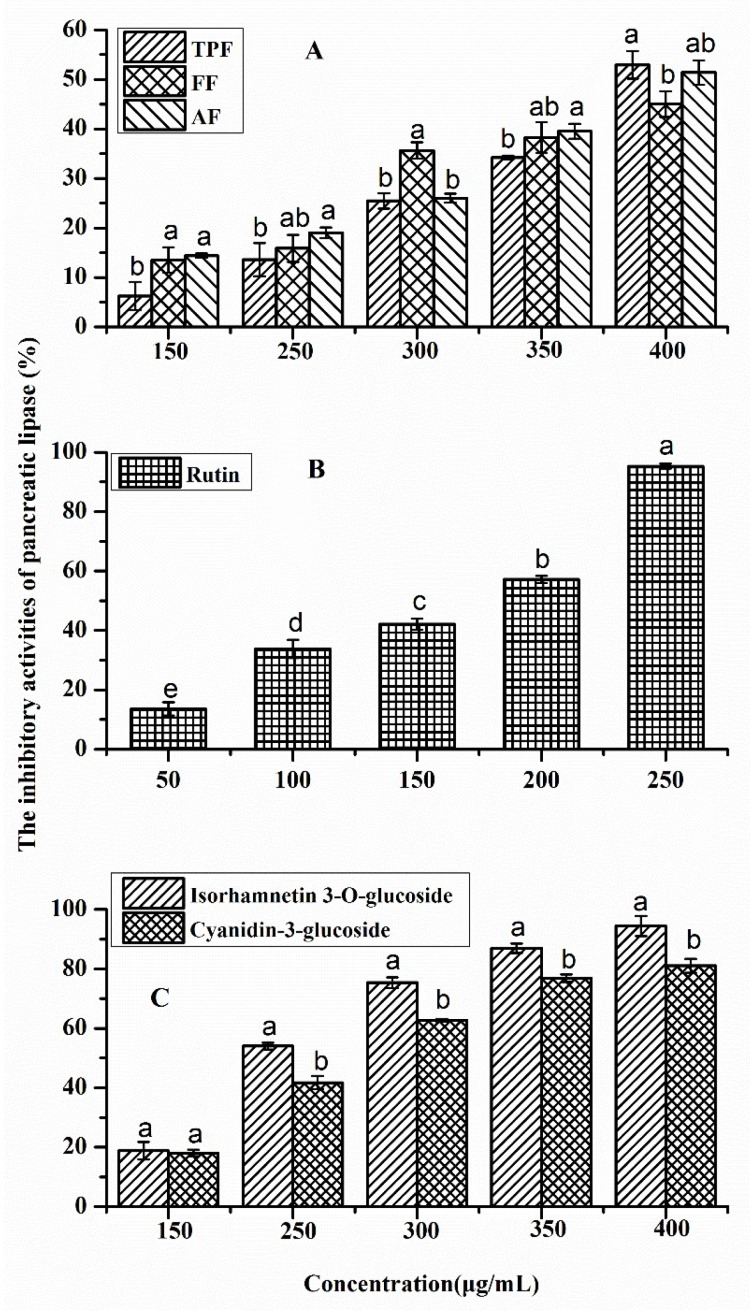
Inhibitory ratio of porcine pancreatic lipase activity by different fractions from *P. utilis* fruits (**A**), rutin (**B**), and isorhamnetin-3-*O*-rutinoside and cyanidin-3-*O*-glucoside (**C**). Values are showed as the mean ± SD (*n* = 3). The means in Figure 3A,C with different letters at the same concentration are significantly different (*p* < 0.05). The means in Figure 3B with different letters are significantly different (*p* < 0.05).

The lipase inhibition results of phenolic fractions from *P. utilis* fruits and three authentic standards are shown in Figure 4. As shown in Figure 4A, all of the fractions exerted good inhibitory effects against pancreatic lipase. The inhibitory rates of these fractions gradually upgraded as their concentrations increased. Although these three fractions had different inhibition ratios of pancreatic lipase at the same tested concentrations, ranging from 150.0 μg/mL to 400.0 μg/mL, the lipase inhibitory capacities of these samples, to some extent, were very close. The IC_50_ values of these fractions revealed a similar result. The IC_50_ values of TPF, FF, and AF were 423.15 ± 11.12 μg/mL, 439.98 ± 25.08 μg/mL, and 407.43 ± 33.89 μg/mL, respectively, which did not have a statistically significant difference from each other (*p* > 0.05). The IC_50_ value of orlistat, which is a lipase inhibitor that was used as positive control, was 317.90 ± 2.37 μg/mL, which was significantly lower than those of the three phenolic fractions (*p* < 0.05). The correlation coefficient between the phenolic contents and lipase inhibition ratios of the three samples at all of the tested concentrations were analyzed by Pearson analysis, the result of which indicated that there was a good positive correlation between these two parameters (r = 0.791, *p* < 0.05), suggesting that phenolics contributed significantly to the lipase capacity of the different phenolic fractions from *P. utilis*. These findings were in consistent with many previous studies [12,13,39,40]. A large number of studies have shown that plant-food extracts that are rich in phenolics often showed a good inhibitory effect against lipase. Zhang et al. [13] reported that all of the phenolic-rich fractions of Chinese sumac (*Rhus chinensis* Mill.) fruits extracted by three different solvents showed similar lipase inhibitory effects, with IC_50_ values ranging from 59.02 μg/mL to 62.88 μg/mL. Another study on the lipase inhibitory effects of the free, esterified, and insoluble-bound phenolic fractions from Chinese sumac fruits found that these different-status phenolic extracts exhibited good and significant different inhibition against lipase, with IC_50_ values varying from 50.12 μg/mL to 142.79 μg/mL [13]. Rahman et al. [39] also reported that all of the phenolic extracts of Camelina and Sophia seeds with differing statuses exerted effective pancreatic lipase inhibition activities, the IC_50_ values of which varied from 4.15 mg to 16.87 mg of extract/mL. According to the IC_50_ values of the aforementioned studies and the current work, it clearly indicated that the phenolic-rich extracts from different plant materials possessed different lipase inhibition capacity, which may be primarily due to their different phenolic compositions.

According to the quantitative results of the identified phenolics in the TPF, FF, and AF of *P. utilis* fruits, rutin, isorhamnetin-3-*O*-glucoside, and cyanidin-3-*O*-glucoside were chosen as the main bioactive compounds for testing their lipase inhibitory effects, and the results are illustrated in Figure 4B,C. The inhibitory ratios of these three phenolic standards also increased with the increased concentrations. The IC_50_ values of rutin, isorhamnetin-3-*O*-rutinoside, and cyanidin-3-*O*-glucoside were 176.18 ± 5.11 μg/mL, 237.87 ± 8.20 μg/mL, and 249.20 ± 3.87 μg/mL, respectively, suggesting that rutin possessed the strongest lipase inhibitory capacity (*p* < 0.05), followed by isorhamnetin-3-*O*-rutinoside and cyanidin-3-*O*-glucoside. Some previous studies on the pancreatic lipase inhibition of various phenolics have also been performed, and the results pointed out that the lipase inhibitory capacity varied profoundly with the phenolic standards [12,13,41,42], which was consistent with the findings of the present work. Those disparities regarding lipase inhibition are most likely caused by the phenolic structural differences.

### 2.4. α-Glucosidase Inhibitory Activities of Phenolic Fractions from P. utilis Fruits and Authentic Standards

**Figure 5:**
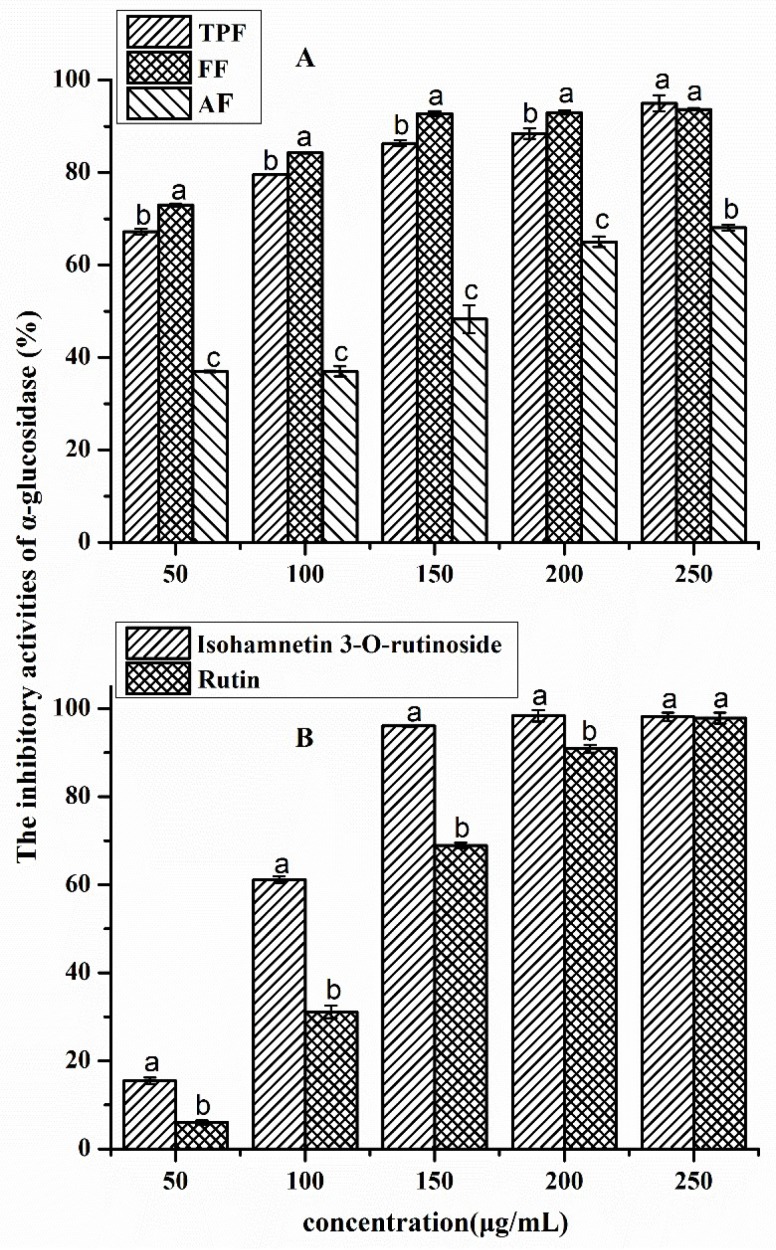
Inhibitory ratio of α-glucosidase activity by different fractions from *P. utilis* fruits (**A**), and isorhamnetin-3-*O*-rutinoside and rutin (**B**). Values are shown as the mean ± SD (*n* = 3). Means with different letters at the same concentration are significantly different (*p* < 0.05).

The results for the α-glucosidase inhibitory activities of different phenolic fractions from *P. utilis* fruits and authentic standards are shown in Figure 5; the three phenolic fractions exhibited α-glucosidase inhibitory effects with a dose-dependent manner, especially the TPF and FF. However, clear differences regarding enzyme inhibition among the three fractions were found. The FF exerted the strongest inhibitory capacity at each concentration ranging from 50.0 μg/mL to 250.0 μg/mL, followed by TPF (*p* < 0.05). The inhibition ratios of those two phenolic fractions were more than 50% at 50.0 μg/mL. However, the AF possessed the weakest inhibition. The IC_50_ values of those three phenolic fractions showed the same trend. The IC_50_ value of the FF was the lowest at 15.93 ± 0.63 μg/mL, while those of the TPF and AF were 20.48 ± 0.37 μg/mL and 149.06 ± 6.34 μg/mL, respectively. The IC_50_ value of acarbose, which is a positive inhibitor of α-glucosidase, was 11.90 ± 0.87 μg/mL; this was significantly lower than those of the three phenolic fractions (*p* < 0.05). Moreover, a clear correlation between the phenolic contents and α-glucosidase inhibitory ratios was observed in the present work (r = 0.754, *p* < 0.05), indicating that the phenolics may be the primary bioactive compounds that are responsible for the α-glucosidase inhibitory effect of this fruits. Many studies in the literature have also reported that phenolic-rich fractions from blueberry [43], black legume [44], camelina seeds, sophia seeds [39], and six millet cultivars [45] possessed good inhibitory effects on α-glucosidase; as a result, the phenolics in those food materials were considered to be the main bioactive compounds. It is worth noting that the phenolic type may be more important than the phenolic content in terms of inhibiting the α-glucosidase, which was partly proved by the current results in Figure 5A and Table 2. The α-glucosidase inhibition ratios of AF at low concentrations (50.0 μg/mL to 150.0 μg/mL) were even less than half those of the inhibition ratios of TPF at the same tested concentration (Figure 5A), even though those two phenolic fractions contained a similar phenolic content (Table 2). The present result indicated that anthocyanins may have a much weaker inhibitory capacity against α-glucosidase than non-anthocyanin phenolics, which was in agreement with the finding of a previous study [43]. Wu et al. [43] reported that the anthocyanin fraction from blueberry had a much weaker α-glucosidase inhibition effect than its copigment fraction (mainly contained non-anthocyanin phenolics).

The rutin and isorhamnetin 3-*O*-rutinoside standards were chosen for evaluating their α-glucosidase inhibition effects because of the above results regarding the quantification and inhibition evaluation, and the results are presented in Figure 5B. The inhibitory ratios also increased with the increased concentrations of these two standards. The isorhamnetin 3-*O*-rutinoside exhibited a significantly stronger inhibition than rutin at each tested concentration (*p* < 0.05), which was in agreement with the results for the IC_50_ values. The IC_50_ values of isorhamnetin 3-*O*-rutinoside and rutin were 90.19 ± 3.27 μg/mL and 126.86 ± 8.58 μg/mL, respectively. Previous studies about the α-glucosidase inhibition of various phenolics have also been studied, and the results also indicated that the α-glucosidase inhibitory capacity was closely dependent on the phenolic standard [46,47]. Han et al. [46] pointed out that phloretin reversibly inhibited α-glucosidase in a mixed-type manner, with an IC_50_ value of 31.26 μg/mL. Wu et al. [47] reported that gallocatechin gallate had strong inhibition on α-glucosidase in a noncompetitive manner, with an IC_50_ value of 30.02 μg/mL. Regardless of the influence of some experimental factors, the α-glucosidase inhibitory capacities of these two phenolics in aforementioned works were stronger than those of the two phenolics in this study.

### 2.5. Molecular Docking Results

According to the current data and the findings of aforementioned works, it is clear that the structures of phenolics profoundly affect their inhibitory effects on digestive enzymes (e.g., pancreatic lipase and α-glucosidase). Therefore, molecular docking analysis was performed by using SYBYL-X 2.1.1 to illuminate the enzyme inhibitory mechanisms of rutin, isorhamnetin-3-*O*-rutinoside, and cyanidin-3-*O*-glucoside. In this work, some relevant parameters (e.g., C-Score, T-Score, PMF-Score, and CHEM-Score) and interactions between the docking sites of enzymes and the phenolic molecules were applied for molecular docking analysis. All of the molecular docking analyses were based on the C-Score results of each phenolic, since the C-Score is considered as an index for molecular docking, and the docking result is credible only when the C-Score ≥ four [13].

**Figure 6:**
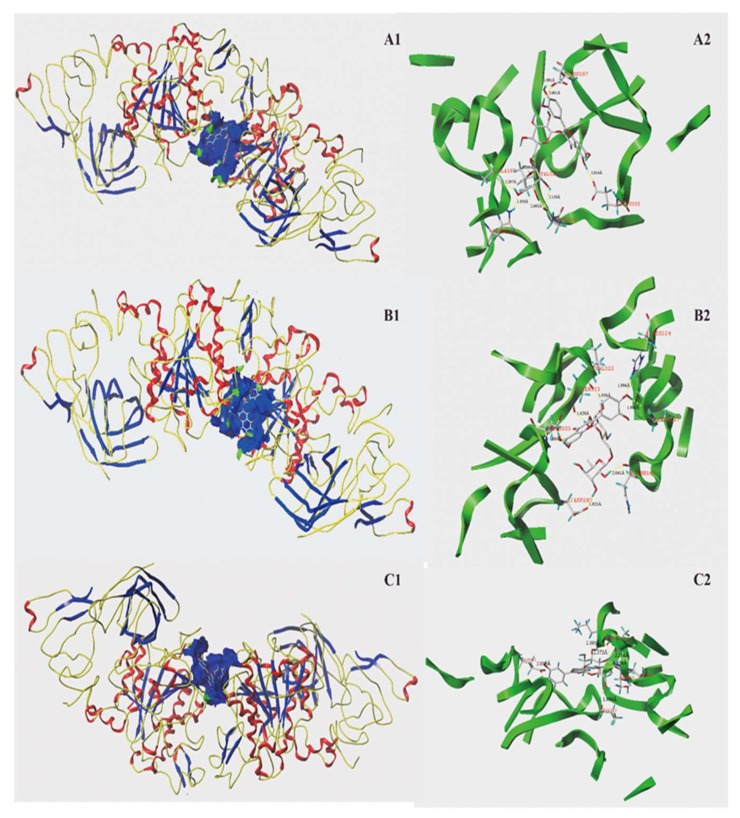
Molecular docking of three phenolic compounds with porcine pancreatic lipase. Three phenolic compounds were inserted into the hydrophobic cavity of porcine pancreatic lipase (blue) in the surface structure ((**A1**), rutin; (**B1**), isorhamnetin-3-*O*-rutinoside; (**C1**), cyanidin-3-*O*-glucoside). Interactions between three phenolic compounds and amino acid residues in the active site of porcine pancreatic lipase: rutin conformation (**A2**), isorhamnetin 3-*O*-rutinoside conformation (**B2**), and cyanidin-3-*O*-glucoside conformation (**C2**), with residues in the active sites of the pancreatic lipase, respectively. The dashed line stands for hydrogen bonds.

The molecular docking results regarding interactions between the three phenolic molecules and pancreatic lipase binding are summarized in Table 3 and Figure 6. As seen in Table 3, the C-Scores of those three phenolics were all no less than four. As shown in Figure 6, rutin interacted with the active site of pancreatic lipase and formed eight H-bonds (dash lines) with six amino acid residues, including Asp 287, Val 322, Ala 197, Asn 320, Glu 303, and His 224. The hydrogen bond distances ranged from 1.850 Å to 2.692 Å. Isorhamnetin-3-*O*-rutinoside formed seven H-bonds with amino acid residues (Val 322, Ser 323, Glu 303, Asp 287, Asn 176, Ala 197, and His 224). The hydrogen bond distances ranged from 1.820 Å to 2.041 Å. Cyandin-3-*O*-glucosidase also formed seven H-bonds with relative amino acid residues within 5 Å of the active site (Glu 303, Ala 197, Ser 195, Lys 198, Val 322, and His 224). The longest and shortest length of these hydrogen bonds were 1.856 Å and 2.395 Å, and the average value was 2.134 Å. All of those three phenolics interacted with the amino acid residues Val322, Ala197, Glu303, and His224 of pancreatic lipase, suggesting that those four amino acid residues may play an important role in the catalytic reaction of lipase. A previous study found that both myricitrin and quercitrin interacted with the amino acid residues Asp 80 and Gly 77 of pancreatic lipase [13]. The different interaction sites between the various phenolics and lipase may explain the differences in the inhibitory capacities of the different phenolics.

**Figure 7:**
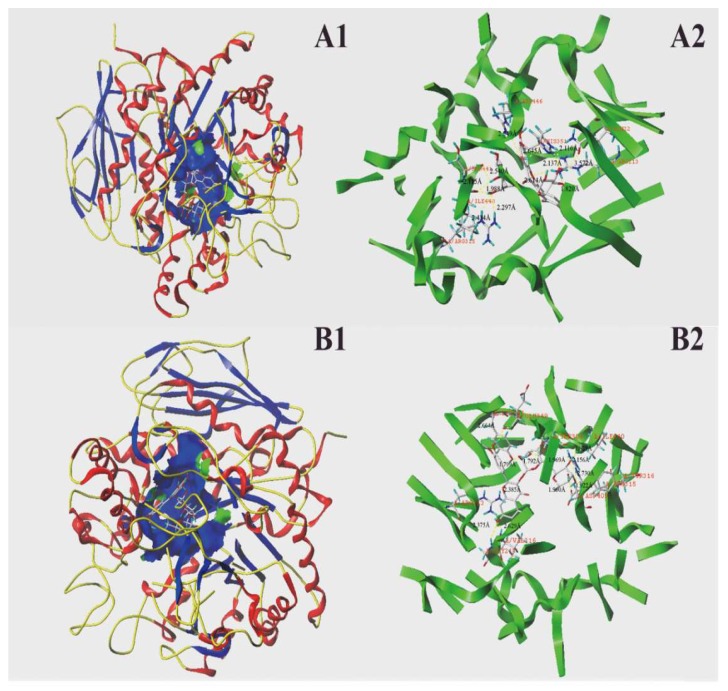
Molecular docking of two phenolic compounds with α-glucosidase. Two phenolic compounds were inserted into the hydrophobic cavity of α-glucosidase (blue) in the surface structure ((**A1**), rutin; (**B1**), isorhamnetin-3-*O*-rutinoside). Interactions between two phenolic compounds and amino acid residues in the active site of α-glucosidase: rutin conformation (**A2**), and isorhamnetin 3-*O*-rutinoside conformation (**B2**), with residues in the active sites of the α-glucosidase, respectively. The dashed line stands for hydrogen bonds.

As for the molecular docking of α-glucosidase, the C-Scores of those two phenolics were also no less than four. Twelve hydrogen bonds (dash lines) were formed between the active site of α-glucosidase and the standards of rutin and isorhamnetin-3-*O*-rutinoside (Figure 7). For rutin, it mainly formed H-bonds with seven amino acid residues with the active site, namely, Gln 22, Ile 440, Ser 441, His 351, Arg 446, Arg 213, and Arg 315. The longest and shortest lengths of these hydrogen bonds were 2.730 Å and 1.799 Å, respectively, and the average value was 2.402 Å. However, isorhamnetin-3-*O*-rutinoside primarily formed H-bonds with 10 amino acid residues that were close to the active site, namely, Tyr 347, Glu 349, Thr 306, Ile 440, Tyr 316, Arg 315, Asp 409, Val 216, Gly 217, and Arg 213. The average length of the hydrogen bonds that were formed between the active sites of α-glucosidase and isorhamnetin-3-*O*-rutinoside was 2.184 Å. The interaction forces of the hydrogen bond are considered to play a critical role in stabilizing the complex of enzyme and ligand in order to exert catalytic reaction, which was mainly dependent on the number and distance between the hydrogen bonds. Although the numbers of hydrogen bonds formed by rutin and isorhamnetin-3-*O*-rutinoside with α-glucosidase were equal, isorhamnetin-3-*O*-rutinoside possessed hydrogen bonds with a much shorter average length, which indicated that isorhamnetin-3-*O*-rutinoside may bind more tightly with α-glucosidase than rutin. This result demonstrated that isorhamnetin-3-*O*-rutinoside has a much stronger inhibitory effect against α-glucosidase, which was consistent with the result obtained by the α-glucosidase inhibition experiment (Figure 5B). Both of those two phenolics interacted with the amino acid residues Ile 440, Arg 213, and Arg 315 of α-glucosidase, indicating that those three amino acid residues may play a critical role in the catalytic reaction of α-glucosidase. Previous studies have pointed out that Val 216, Asp 215, Arg 442, and Glu 411 are believed to be important for the catalytic reaction of α-glucosidase [48,49]. Isorhamnetin-3-*O*-rutinoside also formed a hydrogen bond with Val 216 in the current docking analysis, which indicated that isorhamnetin-3-*O*-rutinoside may bind to the active site of α-glucosidase to inhibit the catalytic activity of this enzyme. Han et al. [46] reported that phloretin also bound to the active site of α-glucosidase, and thereby inhibited the activity of this enzyme in a mix-type manner. Another study pointed out that gallocatechin gallate exhibited a strong inhibition on α-glucosidase in a noncompetitive manner by reacting with Arg 315, Phe 303, and so on [47]. The two phenolics that were used in the current study also formed a hydrogen bond with the amino acid residue of Arg 315 to exert its α-glucosidase inhibitory effect.

## 3. Materials and Methods

### 3.1. Chemicals and Reagents

Methanol, acetonitrile, formic acid, and Folin–Ciocalteu reagent were purchased from Merck (Darmstadt, Germany). Pancreatic lipase (from porcine pancreas, 163 U/mg, EC: 3.1.1.3), α-glucosidase from *Saccharomyces cerevisiae* (Type I, ≥ 10 units/mg protein), p-nitrophenyl–α-D-glucopyranoside (pNPG, purity ≥ 99.0%), acarbose (95%), orlistat (purity ≥ 97.0%), 2,2-Diphenyl-1-picrylhydrazyl (DPPH), 2,2′-azino-bis (3-ethylbenzothiazoline-6-sulphonic acid) (ABTS), p-nitrophenyl laurate (purity ≥ 98.0%), and 2′,7′-Dichlorofluorescin diacetate (DCFH-DA, purity ≥ 97.0% were purchased from Sigma (Sigma-Aldrich, Shanghai, China). Rutin (purity ≥ 98.0%), isorhamnetin-3-*O*-rutinoside (also known as Narcissoside, purity ≥ 98.0%), and quercetin-3-*O*-glucoside (purity ≥ 98.0%) were obtained from Chengdu Must Bio-technology Co., Ltd. (Chengdu, China). All of the other reagents that were used were of analytical grade.

### 3.2. Extraction and Fractionation

Mature fruits (average 0.75 g per fruit) of *P. utilis* were supplied by the Kunming plant-classification biotechnology Co., Ltd. (Kunming, China) in June 2017 collected in Ninglang County (about longitude: 100°45′, latitude: 27°34′), Lijiang City, Yunnan Province and stored at −20 °C for further experiments. Different phenolic fractions of mature fruits were extracted and purified according to the previous reported method [50] with minor modification. Firstly, the seeds in fresh fruits were manually removed, and then the flesh and skin of the fruits were homogenized by an IKA homogenizer (IKA T25 model, IKA Works, Guangzhou, China) at 8000 rpm for two min. The homogenized slurry (50 g, about 140 fruits) was ultrasonically extracted with 200 mL of methanol containing 1% formic acid for 30 min. The extracted mixture was centrifuged for 10 min at 4300× *g*, and the supernatant was filtered. The aforementioned extraction method was repeated twice to the residue. Then, the combined filtrate was evaporated with a rotary evaporator (Hei-VAP, Heidolph, Germany) to remove the organic reagent at 40 °C. Thereafter, the concentrated solution was successively fractionated and purified by an Amberlite XAD-7 column, a Sephadex LH20 column, and an SPE column (Oasis HLB cartridge), as described in the previous study [50]. The eluent from the Amberlite XAD-7 column by acidified methanol (1% formic acid) was evaporated and lyophilized (Alpha 1-2 LD plus, Christ, Germany) as total phenolic fraction (TPF). The eluent from the SPE column by ethyl acetate and acidified methanol (10% formic acid), was evaporated and lyophilized as a flavonoid-rich fraction (FF) and an anthocyanin-rich fraction (AF), respectively. Those fractions were kept at −20 °C until use.

### 3.3. Identification and Quantification of Phenolics by UHPLC-ESI-HRMS/MS

All fractions of *P. utilis* fruits were first separated by using a Thermo Fisher Ultimate 3000 UHPLC System (Thermo Fisher Scientific, Bremen, Germany) equipped with an Agilent Zorbax SB-C18 column (2.1 × 100 mm, 1.7 μm). Acidified water (0.5% formic acid, phase A) and acetonitrile (phase B) were used as mobile phases in the following gradient: 0.01–5 min, 10% B; 5–8 min, 10–15% B; 8–15 min, 15–25% B; 15–18 min, 25% B; 18–19 min, 25–10% B; 19–23 min, 10% B. The injection volume was two μL, the column temperature was set at 35 °C, and the flow rate was 0.2 mL/min. The ESI-HRMS/MS analysis was performed with a Q-Exactive Orbitrap mass spectrometer (Thermo Fisher Scientific, Bremen, Germany) in the negative or positive mode (3.3 kV). The other related parameters were set based on the earlier report [13].

### 3.4. DPPH Radical Scavenging Assay

The antioxidant activities of all of the fractions from *P. utili*s fruits were determined by DPPH assay according to the methods described earlier [32]. The absorbance of the reaction mixture was recorded by a SpectraMax M5 microplate reader (Molecular Device, Sunnyvale, CA, USA) at 517 nm. The DPPH radical scavenging activity (%) was calculated as [(OD_control_ − OD_sample_)/OD_control_] × 100. Each test was measured in triplicate.

### 3.5. ABTS Radical Scavenging Assay

The antioxidant activities of all of the fractions from *P. utilis* fruits were measured by ABTS assay as described earlier [32]. The absorbance of the reaction mixture was determined with a SpectraMax M5 microplate reader at 745 nm. The ABTS radical scavenging activity (%) of each sample was calculated as [(OD_control_ − OD_sample_)/OD_control_] × 100. Each test was measured three times.

### 3.6. Determination of Intracellular ROS

HepG2 cells were supplied by Kunming cell bank, Chinese Academy of Sciences (Kunming, China), which were cultured in normal Dulbecco’s Modified Eagle Medium (DMEM) medium containing 10% fetal bovine serum (FBS), 1% antibiotic mixture of streptomycin (100 mg/mL) and penicillin (100 U/mL) at 37 °C with a humidified 5% CO_2_ atmosphere. The methylthiazolyldiphenyl-tetrazolium bromide (MTT) assay was applied to evaluate the cytotoxicity of the sample to HepG2 cells [32]. All of the samples exhibited no toxicity to HepG2 cells at all of the tested concentrations that were used in this work. The ROS detection method referenced the earlier literature [50] with minor modifications. Briefly, cells were seeded on six-well plates at 2.0 × 10^5^ cells per well, and incubated for 24 h. Thereafter, DMEM with or without tested samples was added into each well and removed at the end of the incubation period (24 h), followed by being attacked with one mM of H_2_O_2_ for four h (except for the control group), and each group had three repetitions. Then, cells were harvested and washed twice with phosphate buffer solution (PBS). The cells of all of the groups were incubated with 10 μM of DCFH-DA for 20 min, and washed with serum-free medium to remove excess DCFH-DA by centrifuging at 156× *g* for five minutes. Subsequently, the intracellular ROS of each group was measured by flow cytometry with guava^®^ easyCyte 6-2L (Millipore, Billerica, MA, USA) within 45 min. All of the experiments were measured in triplicate.

### 3.7. Determination of Pancreatic Lipase Inhibition

The inhibitory effects of different fractions from *P. utilis* fruits, rutin, isorhamnetin-3-*O*-glucoside, and cyanidin-3-*O*-glucoside against pancreatic lipase activity were measured according to the method reported earlier [13]. The absorbance (OD) was measured at 400 nm by using a SpectraMax M5 microplate reader. Each test was measured three times.

### 3.8. Determination of α-Glucosidase Inhibition

The α-glucosidase inhibition activities of different fractions from *P. utilis* fruits, rutin, and isorhamnetin-3-*O*-glucoside were evaluated as previously described [51] with some modifications. The absorbance (OD) of the each reaction mixture at 405 nm was recorded by using a SpectraMax M5 microplate reader (Molecular Device, Sunnyvale, CA, USA).

### 3.9. Molecular Docking

The molecular docking assays of three predominant phenolic standards (rutin, isorhamnetin-3-*O*-glucoside, and cyanidin-3-*O*-glucoside) and orlistat (a pancreatic lipase inhibitor) to pancreatic lipase, and two phenolic standards (rutin and isorhamnetin-3-*O*-glucoside) and acarbose (α-glucosidase inhibitor) to α-glucosidase, were performed by using SYBYL-X 2.1.1 (Tripos, Inc., St. Louis, MO, USA), according to previous studies [13,46,52]. The three-dimensional (3D) conformers of the phenolic standards and two-dimensional (2D) structures of orlistat and acarbose were downloaded from PubChem (https://www.ncbi. nlm.nih.gov/pccompound), and the PDB format of the porcine pancreatic lipase (PDB ID:1ETH) and isomaltase (PDB ID: 3A4A) was downloaded from RCSB PDB (http://www.rcsb.org/pdb/home/home.do). The homology modeling of α-glucosidase (*S. cerevisiae*) was performed by SYBYL-X 2.1.1 due to the structural information of α-glucosidase (*S. cerevisiae*) not being available. The homology modeling result showed that isomaltase from the same organism shares high similarity with α-glucosidase. Therefore, isomaltase was used as the template in the current α-glucosidase docking analysis. The Surflex-Dock Geom (SFXC) was used as the docking mode. All of the water substructures were deleted in the “remove substructures” option. Hydrogens (Random) and charges (Gasteiger–Huckel) were added. Subsequently, a lipase–ligand file was generated and saved as a molecular SD format. C-Score calculations were applied during the binding procedure in order to select the better results for analysis.

### 3.10. Statistical Analysis

Data are presented as mean values (*n* = three) ± standard deviation (SD) and analyzed by using one-way ANOVA. Tukey’s procedure was applied to determine the significance of differences (*p* < 0.05). All of the analyses were performed by using Origin 8.5 software (OriginLab, Northampton, MA, USA). The Pearson’s correlation coefficient was examined by using SPSS 20.0 (SPSS, Inc., Chicago, IL, USA).

## 4. Conclusions

In the present study, the phenolic composition in the TPF, FF, and AF from *P. utilis* fruits was analyzed by UHPLC-ESI-HRMS/MS. A total of 20 phenolics were identified and quantified. Rutin, quercetin-3-*O*-glucoside, and isorhamnetin-3-*O*-rutinoside were the major phenolic compounds in the TPF and FF. The AF mainly contained cyanidin-3-*O*-glucoside and cyanidin-3-*O*-rutinoside. All of the fractions showed good radical scavenging activities and the strong inhibition of cellular ROS generation of in H_2_O_2_-induced HepG2 cells. Meanwhile, those fractions exhibited powerful inhibitory effects against pancreatic lipase and α-glucosidase. The major phenolic compounds that were detected in those fractions also exerted good digestive enzymes inhibitory activities in a concentration-dependent manner. Molecular docking analysis revealed the underlying inhibition mechanisms of those phenolic standards against digestive enzymes, which were consistent with the experimental results.

## Figures and Tables

**Table 1:** Main chemical compounds detected and characterized of the different phenolic fractions of *P. utilis* fruits using UHPLC-ESI-HRMS/MS in negative/positive mode.

Peak No.	Compounds	RT ^a^ (min)	[M − H]^−^ (*m/z*)	[M]^+^ (*m/z*)	Error (ppm)	Molecular Formula	MS/MS Fragment Ions	Reference	TPF *	FF *	AF *
1	Protocatechuic acid	3.68	153.0187		2.907	C_7_H_6_O_4_	109.0284(66)	Standard	√	√	
2	Penstemide	4.77	443.1935		5.272	C_21_H_32_O_10_	101.0233(90)	[23]	√		
3	p-Coumaric acid	6.43	163.0394		0.495	C_9_H_8_O_3_	119.0492(100)	Standard	√	√	
4	Kaempferol-3-*O*-glucoside	8.96	447.0946		5.328	C_21_H_20_O_1_1	284.0333(100), 285.0408(60)	Standard	√	√	
5	Kaempferol-3-*O*-rhamnosylhexose	10.32	593.1531		5.131	C_27_H_30_O_15_	284.0328(10), 593.1530(100)	[24]	√	√	
6	Dihydroquercetin rhamnoside	10.88	449.1102		5.326	C_21_H_22_O_11_	125.0235(61), 151.0030(20)	[24]	√	√	
7	Catechin	10.92	289.0726		6.695	C_15_H_14_O_6_	123.0442(80), 109.0284(100)	Standard	√	√	
8	Isoschaftoside	13.56	563.1422		4.649	C_26_H_28_O_14_	563.1422(100), 353.0677(61)	Standard	√	√	
9	Rutin	15.42	609.1476		4.217	C_27_H_30_O_16_	300.0282(77), 609.1479(100)	Standard	√	√	√
10	Quercetin-3-*O*-glucoside	16.01	463.0896		5.329	C_21_H_20_O_12_	271.0254(30), 300.0282(100)	Standard	√	√	√
11	Quercetin 3-(6-*O*-acetyl-beta-glucoside)	16.92	505.1003		5.173	C_23_H_22_O_13_	300.0283(100), 301.0351(45)	Mass bank	√	√	
12	Isorhamnetin-3-*O*-rutinoside	17.39	623.1636		4.668	C_28_H_32_O_16_	315.0519(100), 623.1638(38)	Standard	√	√	√
13	Kaempferol-3-*O*-hexoside	17.63	447.0946		5.328	C_21_H_20_O_11_	285.0395(30), 284.0334(100)	[24]	√	√	
14	Isorhamnetin-3-*O*-glucoside	18	477.1051		4.858	C_22_H_22_O_12_	314.0441(100), 477.1050(20)	Standard	√	√	√
15	Cyanidin-3-*O*-glucoside	9.98		449.1074	−1.398	C_21_H_21_O_11_	287.0548(100), 288.0580(17)	Standard	√	√	√
16	Cyanidin-3-*O*-rutinoside	10.91		595.1655	−1.086	C_27_H_31_O_15_	287.0549(100), 449.1072(10)	[25]	√	√	√
17	Peonidin-3-*O*-rutinoside	12.87		609.1810	−0.700	C_28_H_33_O_15_	301.0705(100), 463.1230(10)	[25]	√		√
18	Peonidin-3-*O*-sophoroside-5-*O*-glucoside	13.38		787.2283	−1.251	C_34_H_43_O_21_	463.1229(10)	[26]	√		√
19	Delphinidin-3-*O*-rutinoside	14.71		611.1603	−0.722	C_27_H_31_O_16_	303.049(100), 304.0528(20)	Mass bank	√		√
20	Petunidin-3-*O*-glucoside	16.83		479.1178	−1.070	C_22_H_23_O_12_	317.0653(100)	[25]	√		√

^a^ RT, retention time; * TPF means the total phenolic fraction; FF means flavonoid fraction; AF means anthocyanin fraction.

**Table 2:** Quantitative result of the identified phenolics in the different phenolic fractions (TPF, FF, and AF) of *P. utilis* fruits by UPLC-ESI-HRMS/MS ^a^.

Peak No.	Compounds	TPF (μg/g)	FF (μg/g)	AF (μg/g)
1	Protocatechuic acid	861.69 ± 23.72d	297.66 ± 33.54c	ND *
3	Coumaric acid	1354.42 ± 97.94d	1459.71 ± 94.02c	ND
4	Kaempferol-3-*O*-glucoside ^b^	218.02 ± 34.34d	4195.15 ± 116.62c	ND
5	Kaempferol-3-*O*-rhamnosylhexose ^b^	131.91 ± 20.82d	1909.04 ± 78.39c	ND
6	Dihydroquercetin rhamnoside ^b^	302.07 ± 1.13c	Trace	ND
7	Catechin	486.50 ± 9.92d	3258.80 ± 119.01c	ND
8	Isoschaftoside	645.95 ± 25.38c	67.65 ± 6.65c	ND
9	Rutin	10,177.90 ± 468.53c	5082.50 ± 200.46d	Trace
10	Quercetin-3-*O*-glucoside ^b^	1274.17 ± 98.72d	8685.38 ± 179.22c	Trace
11	Quercetin 3-(6-*O*-acetyl-beta-glucoside) ^b^	63.10 ± 7.07d	189.02 ± 12.70c	ND
12	Isorhamnetin-3-*O*-rutinoside	7072.17 ± 27.93c	7566.58 ± 503.86c	Trace
13	Kaempferol-3-*O*-hexoside ^b^	44.01 ± 0.05d	459.90 ± 21.08c	ND
14	Isorhamnetin-3-*O*-glucoside	566.02 ± 17.17d	7443.42 ± 119.55c	Trace
15	Cyanidin-3-*O*-glucoside	2806.80 ± 159.50	Trace	15,800.42 ± 822.41c
16	Cyanidin-3-*O*-rutinoside ^b^	1003.42 ± 347.20	Trace	5011.44 ± 282.87c
17	Peonidin-3-*O*-rutinoside ^b^	Trace	ND	684.64 ± 65.97c
18	Peonidin-3-*O*-sophoroside-5-glucoside ^b^	Trace	ND	192.08 ± 18.51c
19	Delphinidin-3-*O*-rutinoside ^b^	Trace	ND	639.78 ± 61.64c
20	Petunidin-3-*O*-glucoside ^b^	Trace	ND	246.67 ± 23.77c
Total content		27,008.15	40,614.81	22,575.03

^a^ TPF means the total phenolic fraction; FF means flavonoid fraction; AF mean anthocyanin fraction; Values were expressed as the mean ± SD (*n* = 3). Means in the same row without a lowercase letter in common differ significantly (*p* < 0.05). The results were expressed as μg/g of dry extract; ^b^ These 11 specific phenolics were quantified by kaempferol-7-*O*-glucoside, kaempferol-7-*O*-glucoside, quercitrin, quercitrin, quercitrin, kaempferol-7-*O*-glucoside, cyanidin-3-*O*-glucoside, cyanidin-3-*O*-glucoside, cyanidin-3-*O*-glucoside, cyanidin-3-*O*-glucoside, and cyanidin-3-*O*-glucoside, respectively; * ND means not detected.

**Table 3:** The analysis results of the molecular dockings into pancreatic lipase or α-glucosidase ligands.

		C-Score	T-Score	PMF-Score	CHEM-Score	G-Score	D-Score
**Lipase**	Rutin	4	8.3565	17.9959	−13.5442	−186.0455	−186.0455
	Isorhamnetin-3-*O*-rutinoside	5	5.7784	9.6420	−18.6390	−171.0586	−175.6447
	Cyanidin-3-*O*-glucoside	4	5.5067	−3.5225	−17.2561	−196.237	−118.6521
**α-Glucosidase**	Rutin	5	7.8139	−127.513	−21.6289	−56.3033	−155.2835
	Isorhamnetin 3-*O*-rutinoside	5	7.1493	−106.7521	−27.5	−201.3377	−225.5207
